# Supplementary material for: Modeling glioblastoma heterogeneity as a dynamic network of cell states
Source: Mol Syst Biol. 2021 Sep 16;17(9):e10105. doi: 10.15252/msb.202010105 (PMC8444284; doi:10.15252/msb.202010105)
Supplement: Supplementary file 6 — Source Data for Figure 5 [file MSB-17-e10105-s004.zip › Figure5A_sourcedata/GSEA_3071/hallmarks_stateI.GseaPreranked.1623742531165/HALLMARK_BILE_ACID_METABOLISM.html]

Details for gene set HALLMARK\_BILE\_ACID\_METABOLISM[GSEA]

|  || Dataset | state13071 |
| Phenotype | NoPhenotypeAvailable |
| Upregulated in class | na\_pos |
| GeneSet | HALLMARK\_BILE\_ACID\_METABOLISM |
| Enrichment Score (ES) | 0.5103582 |
| Normalized Enrichment Score (NES) | 1.8865801 |
| Nominal p-value | 0.004651163 |
| FDR q-value | 0.007868469 |
| FWER p-Value | 0.054 |
Table: GSEA Results Summary

  

Fig 1: Enrichment plot: HALLMARK\_BILE\_ACID\_METABOLISM      
 Profile of the Running ES Score & Positions of GeneSet Members on the Rank Ordered List

  

| PROBE | GENE SYMBOL | GENE\_TITLE | RANK IN GENE LIST | RANK METRIC SCORE | RUNNING ES | CORE ENRICHMENT || 1 | SLC29A1 |  |  | 50 | 1.357 | 0.1306 | Yes |
| 2 | GCLM |  |  | 161 | 0.891 | 0.1943 | Yes |
| 3 | DIO2 |  |  | 212 | 0.803 | 0.2658 | Yes |
| 4 | OPTN |  |  | 339 | 0.637 | 0.2978 | Yes |
| 5 | ABCA1 |  |  | 345 | 0.633 | 0.3640 | Yes |
| 6 | BCAR3 |  |  | 354 | 0.628 | 0.4287 | Yes |
| 7 | ABCA5 |  |  | 465 | 0.538 | 0.4547 | Yes |
| 8 | PEX11A |  |  | 639 | 0.439 | 0.4521 | Yes |
| 9 | CROT |  |  | 739 | 0.400 | 0.4666 | Yes |
| 10 | HSD17B11 |  |  | 779 | 0.389 | 0.4970 | Yes |
| 11 | SCP2 |  |  | 868 | 0.361 | 0.5104 | Yes |
| 12 | GSTK1 |  |  | 1113 | 0.304 | 0.4730 | No |
| 13 | IDH2 |  |  | 1324 | 0.264 | 0.4412 | No |
| 14 | EFHC1 |  |  | 1614 | -0.269 | 0.3873 | No |
| 15 | RBP1 |  |  | 3091 | -0.667 | 0.0363 | No |
| 16 | PXMP2 |  |  | 3200 | -0.782 | 0.0890 | No |
Table: GSEA details [plain text format]

  

Fig 2: HALLMARK\_BILE\_ACID\_METABOLISM: Random ES distribution      
 Gene set null distribution of ES for **HALLMARK\_BILE\_ACID\_METABOLISM**
